# Risk Factors for Bleeding Following Combined Catheter Ablation and Left Atrial Appendage Occlusion in Patients With Nonvalvular Atrial Fibrillation and Low Baseline Bleeding Risk: An Exploratory Analysis

**DOI:** 10.7759/cureus.105313

**Published:** 2026-03-16

**Authors:** Haoqing Ren, Hengli Lai, Zhenhuan Chen

**Affiliations:** 1 Department of Cardiology, Jiangxi Medical College, Nanchang University, Nanchang, CHN; 2 Department of Cardiology, Jiangxi Provincial People's Hospital, Nanchang, CHN

**Keywords:** atrial fibrillation, bleeding risk, catheter ablation, left atrial appendage occlusion, risk factors

## Abstract

Background and objective: In patients with nonvalvular atrial fibrillation (NVAF) undergoing combined catheter ablation (CA) and left atrial appendage occlusion (LAAO), bleeding events may occur even among those classified as low risk by the HAS-BLED score (≤2). This exploratory study aimed to identify factors associated with post-procedural bleeding in this specific population.

Methods: This single-center retrospective analysis included 209 NVAF patients with HAS-BLED ≤2 who underwent first-time CA and LAAO between 2021 and 2023. Patients with active bleeding or severe hepatic/renal impairment (estimated glomerular filtration rate (eGFR) <30 mL/min/1.73 m²) were excluded. The primary outcome was any Bleeding Academic Research Consortium (BARC)-defined bleeding event over a mean follow-up of 30 months. Bleeding severity (BARC 1, ≥2, ≥3) and timing (periprocedural (≤30 days) vs. late) were characterized. Given the limited number of bleeding events (n=10), multivariable analysis employed Firth's penalized-likelihood regression to reduce small-sample bias. Model performance was assessed using the c-statistic with optimism-corrected bootstrap validation.

Results: Ten patients (4.78%) experienced bleeding events (incidence rate: 1.96 per 100 person-years). Bleeding was predominantly BARC ≥2 (n=8, 80%), with five events (50%) occurring periprocedurally. In univariable analysis, age (OR 1.17 per year, 95% CI 1.05-1.30), CHA₂DS₂-VASc score (OR 2.04, 95% CI 1.28-3.26), and renal insufficiency (eGFR <60 mL/min/1.73 m²; OR 23.87, 95% CI 5.73-99.49) were associated with bleeding. In multivariable Firth regression, age (adjusted OR (aOR) 1.13 per year, 95% CI 1.03-1.28; absolute risk difference per 10-year increase: +5.2%), history of myocardial infarction (MI) (aOR 36.82, 95% CI 2.62-446.55; absolute risk difference: +41.3%), and renal insufficiency (aOR 21.16, 95% CI 4.50-106.48; absolute risk difference: +35.8%) remained independently associated with bleeding. The optimism-corrected c-statistic was 0.82 (95% CI 0.71-0.91). However, with only 10 events and three predictors examined, the analysis is susceptible to overfitting, and effect estimates, particularly for MI, have limited precision.

Conclusion: In this exploratory, hypothesis-generating analysis of NVAF patients with low HAS-BLED scores undergoing combined CA and LAAO, renal insufficiency, history of MI, and advanced age were associated with bleeding events. These findings suggest that conventional risk scores may not fully capture bleeding risk in this setting but require validation in larger, prospective multicenter cohorts before clinical application.

## Introduction

The global burden of atrial fibrillation (AF) continues to increase, with prevalence rising from 33.5 million to 59.0 million between 2010 and 2019 [1]. It elevates stroke risk four- to fivefold [2], with the left atrial appendage (LAA) serving as the predominant thrombus source in nonvalvular AF (NVAF) [3]. While oral anticoagulation effectively reduces stroke risk [4], its utility may be limited by contraindications, high bleeding risk, or patient preference [5,6]. For such patients, left atrial appendage occlusion (LAAO) has emerged as a validated alternative [7], and catheter ablation (CA) offers effective rhythm control for symptomatic AF [8-10].

The combined 'one-stop' CA and LAAO procedure has been increasingly adopted, with accumulating evidence supporting its feasibility and safety [7,11,12]. This approach offers the theoretical advantage of addressing both rhythm control and stroke prevention in a single intervention while potentially reducing long-term anticoagulation requirements [13,14].

Current evidence regarding bleeding risk after combined procedures derives predominantly from studies of patients at high bleeding risk. Consequently, risk factors for bleeding among patients classified as low risk by the HAS-BLED score (≤2) remain poorly characterized. This knowledge gap is clinically relevant because (1) the HAS-BLED score was developed to predict bleeding during oral anticoagulation, not the unique peri-procedural risk state created by invasive procedures and post-procedural antithrombotic therapy; (2) threshold-based scoring (e.g., age >65, 'severe' renal impairment) may miss clinically important risk gradients; and (3) procedure-related factors may modify bleeding risk independent of baseline clinical characteristics [15]. Therefore, this exploratory study aimed to identify factors associated with bleeding events following combined CA and LAAO in NVAF patients with low baseline HAS-BLED scores, to generate hypotheses for future prospective risk modeling.

## Materials and methods

Study population

This single-center retrospective analysis initially identified 217 patients from the Chinese Atrial Fibrillation Center registry and the electronic medical record system of Jiangxi Provincial People's Hospital (Nanchang, JX, CHN). The study was approved by the Medical Ethics Committee of Jiangxi Provincial People's Hospital (approval no. 76). After applying the exclusion criteria, 209 patients were ultimately enrolled. To reflect real-world practice, the study cohort also included patients with off-label device use or contraindications to anticoagulation.

Inclusion criteria were a diagnosis of NVAF per European Society of Cardiology/American College of Cardiology guidelines, a HAS-BLED score of ≤2, a first-time combined CA and LAAO procedure, and age ≥18 years. Exclusion criteria were valvular AF, a previous history of LAAO or CA, severe hepatic or renal impairment (defined as Child-Pugh class C liver function or estimated glomerular filtration rate (eGFR) <30 mL/min/1.73 m²), active bleeding, malignancy, or loss to follow-up or incomplete clinical data.

Procedural details

Catheter ablation was performed using radiofrequency energy (ThermoCool SmartTouch SF; Biosense Webster, Irvine, CA, USA) in all cases. The ablation strategy included circumferential pulmonary vein isolation, with additional linear lesions or complex fractionated electrogram ablation at the operator's discretion. The LAAO was performed using Watchman FLX (Boston Scientific, Marlborough, MA, USA) or Amplatzer Amulet (Abbott Laboratories, Lake Bluff, IL, USA) devices. Device selection and sizing were determined by intra-procedural transesophageal echocardiography and fluoroscopic guidance. All procedures were performed under general anesthesia with intraprocedural heparin to maintain activated clotting time >300 seconds.

Periprocedural and postprocedural antithrombotic management

The periprocedural antithrombotic protocol followed institutional guidelines. Anticoagulation (direct oral anticoagulants (DOACs) or warfarin) was interrupted 24 to 48 hours pre-procedure, depending on renal function, and resumed 48 hours post-procedure after confirmation of no access site or pericardial bleeding. Post-discharge, patients received either (1) DOAC monotherapy (dabigatran 110 mg twice a day (BID) or rivaroxaban 15 mg daily, adjusted for renal function) or (2) warfarin (target INR 2.0-3.0) for 45 days, followed by dual antiplatelet therapy (aspirin 100 mg daily + clopidogrel 75 mg daily) for six months and aspirin monotherapy thereafter. The specific regimen was determined by the treating physician based on bleeding risk assessment and device type.

Data collection

Baseline clinical characteristics and laboratory data during the index hospitalization were collected from the hospital's AF center database and electronic medical records. Follow-up data were obtained via outpatient reviews, telephone interviews, or readmission records over a mean follow-up period of 30 months. Collected variables included age, sex, CHA₂DS₂-VASc score [15], HAS-BLED score, comorbidities, renal insufficiency (defined as an eGFR <60 mL/min/1.73 m², corresponding to chronic kidney disease (CKD) stage G3 or greater), smoking history, alcohol use, serum creatinine level, and N-terminal pro-B-type natriuretic peptide level. The occurrence of bleeding events during follow-up was meticulously recorded. The CHA₂DS₂-VASc score was calculated as described by Lip et al. [15], and the HAS-BLED score was calculated per Pisters et al. [16].

Clinical outcomes

The primary outcome was the occurrence of any bleeding event during the 30-month follow-up period. Bleeding events were classified according to the Bleeding Academic Research Consortium (BARC) criteria (2011) [17]: grade 0, no bleeding; grade 1, minor bleeding requiring no intervention; grade 2, measurable bleeding with hemoglobin decrease ≥20 g/L, but no transfusion or intervention required; grade 3, clinically significant bleeding with hemoglobin decrease ≥30 g/L, requiring transfusion or interventional hemostasis, or accompanied by hemodynamic instability, intracranial hemorrhage (excluding minor bleeding), or intraocular hemorrhage affecting vision; grade 4, coronary artery bypass graft-related bleeding; grade 5, fatal bleeding. Bleeding severity was categorized as BARC 1 (minor), BARC ≥2 (clinically relevant), and BARC ≥3 (severe). Bleeding timing was classified as periprocedural (≤30 days post-procedure) or late (>30 days). Events were adjudicated by two independent physicians blinded to patient characteristics, with disagreements resolved by consensus.

Statistical analysis

Statistical analyses were performed using R Studio version 4.5.1 (R Project for Statistical Computing, R Foundation, Vienna, AUT). Continuous variables are presented as mean ± standard deviation or median (interquartile range), and categorical variables as frequencies (percentages). Univariate analyses were conducted using the chi-square test (or Fisher's exact test where appropriate) for categorical variables and the Student's t-test for continuous variables. Incidence rates were calculated as events per 100 person-years.

Univariate logistic regression was employed to identify potential risk factors. Given the exploratory nature of the study and the limited number of bleeding events (n=10), a conservative analytic approach was adopted. Variables with a p-value <0.1 in the univariate analysis were considered for multivariable modeling, with the following a priori constraints: given 10 events, examination of >3-4 predictors risked overfitting (events-per-variable (EPV) <3). Therefore, the primary multivariable model was limited to three predictors based on clinical plausibility and univariable signal strength.

Given the small number of bleeding events (n=10), which can lead to biased estimates and unreliable confidence intervals in traditional maximum-likelihood logistic regression, multivariable analysis was performed using Firth's penalized-likelihood logistic regression [18,19]. This method introduces a penalty based on the Fisher information matrix, effectively reducing small-sample bias and providing more stable estimates.

Model performance and absolute risk differences

Discrimination was assessed using the c-statistic (area under the receiver operating characteristic curve). To evaluate optimism and potential overfitting, internal validation was performed using 200 bootstrap samples, with calculation of the optimism-corrected c-statistic. For each independent predictor, absolute risk differences were calculated as the difference in predicted probability of bleeding between patients with vs. without the risk factor, holding other covariates at their mean values.

Collinearity assessment and sensitivity analyses

Variance inflation factors (VIFs) were calculated for all predictors in the multivariable model. Given the conceptual overlap between age and CHA₂DS₂-VASc (which includes age as a component), the correlation between these variables was examined using Pearson's correlation coefficient. To assess the robustness of the findings, we performed (1) analysis limited to clinically relevant bleeding (BARC ≥2) as the outcome; (2) examination of bleeding incidence stratified by CKD stage; (3) assessment of model stability with and without inclusion of extreme-influence cases (e.g., single myocardial infarction (MI) event) via influence diagnostics.

## Results

Study population and baseline characteristics

**Figure 1:**
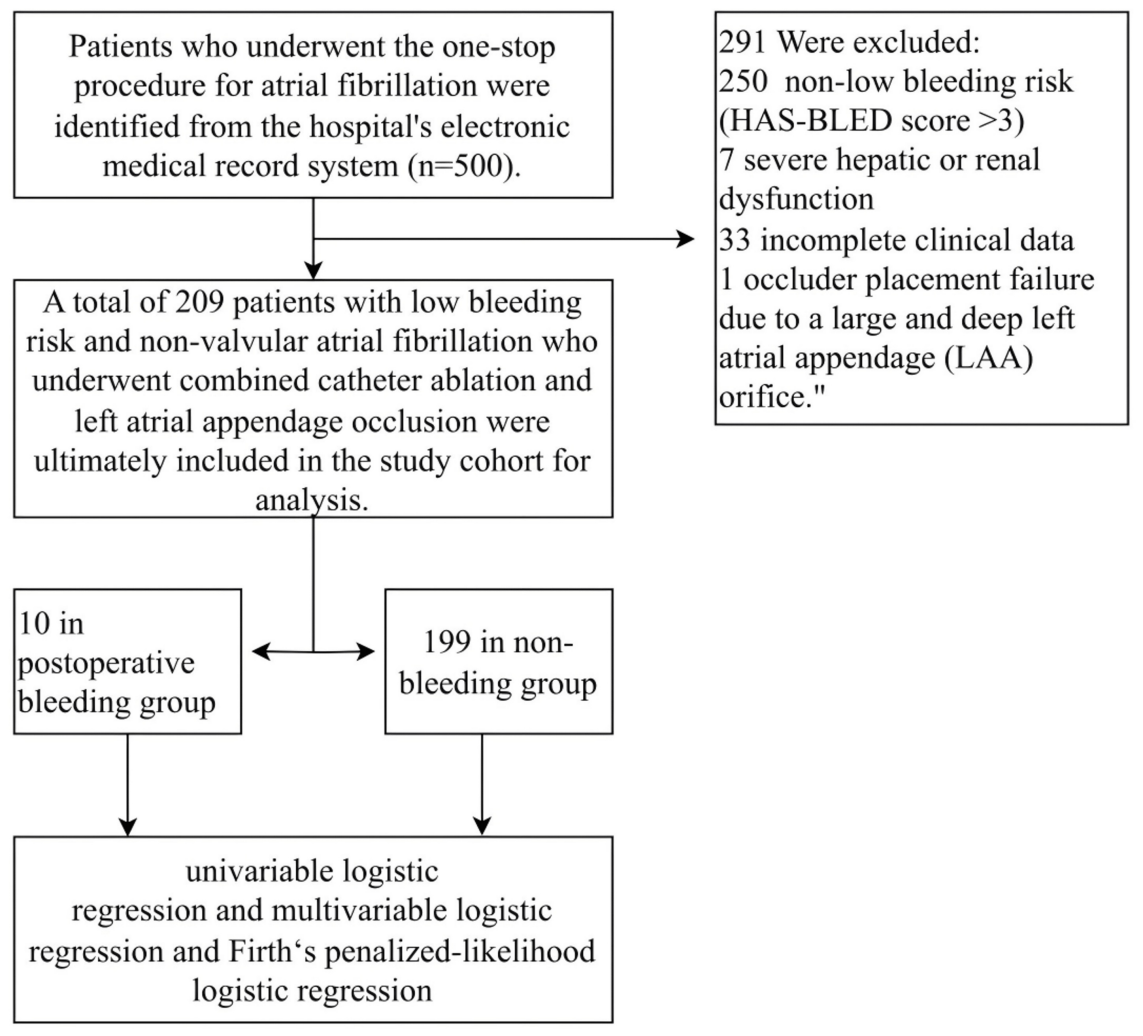
Flowchart detailing patient enrolment

A total of 500 patients who underwent the one-stop AF procedure were initially screened. After applying the inclusion and exclusion criteria, the final analysis cohort consisted of 209 patients. Among them, 10 patients (4.78%) experienced postoperative bleeding events, while 199 patients (95.22%) did not (Figure 1). This corresponds to an incidence rate of 1.96 per 100 person-years.

Baseline characteristics are summarized in Table 1. Significant differences were observed between the bleeding and non-bleeding groups regarding age (75.10 ± 8.29 vs. 66.55 ± 8.66 years, p=0.003), CHA₂DS₂-VASc score (5.10 ± 1.37 vs. 3.69 ± 1.32, p=0.001), and the prevalence of renal insufficiency (50.0% vs. 4.0%, p<0.001). Mean eGFR was significantly lower in the bleeding group (52.4 ± 15.3 mL/min/1.73m² vs. 78.6 ± 14.2 mL/min/1.73m², p<0.001). No significant differences were found in other variables, including hypertension, diabetes, type of AF, or postoperative anticoagulation regimen.

**Table 1:** Baseline characteristics of patients stratified by postoperative bleeding events ^†^Student's t-test; ^‡^Fisher's exact test; ^§^Chi-square test; TIA: Transient ischemic attack; MI: Myocardial infarction; eGFR: Estimated glomerular filtration rate

Characteristics		Overall (n=209)	No bleeding (n=199)	Bleeding (n=10)	p-value
Demographics	Age, years, mean ± SD	66.96 ± 8.82	66.55 ± 8.66	75.10 ± 8.29	0.003^†^
	Male sex, n (%)	114 (54.5)	109 (54.8)	5 (50.0)	1.000^‡^
Risk scores, mean ± SD	CHA₂DS₂-VASc	3.76 ± 1.36	3.69 ± 1.32	5.10 ± 1.37	0.001^†^
Comorbidities, n (%)	Heart failure	121 (57.9)	114 (57.3)	7 (70.0)	0.641^‡^
	Hypertension	128 (61.2)	120 (60.3)	8 (80.0)	0.360^‡^
	Diabetes mellitus	34 (16.3)	31 (15.6)	3 (30.0)	0.443^‡^
	History of transient ischemic attack (TIA)/stroke	81 (38.8)	74 (37.2)	7 (70.0)	0.081^‡^
	MI	3 (1.4)	2 (1.0)	1 (10.0)	0.137^‡^
	Renal insufficiency (eGFR <60)	13 (6.2)	8 (4.0)	5 (50.0)	<0.001^‡^
	eGFR, mL/min/1.73m², mean ± SD	77.2 ± 15.1	78.6 ± 14.2	52.4 ± 15.3	<0.001†
Postoperative antithrombotic regimen, n (%)	Dabigatran	76 (36.4)	72 (36.2)	4 (40.0)	0.265^§^
	Warfarin	6 (2.9)	5 (2.5)	1 (10.0)	
	Rivaroxaban	127 (60.8)	122 (61.3)	5 (50.0)	

Bleeding event characteristics

Among the 10 bleeding events, severity distribution was BARC 1 (n=2, 20%), BARC 2 (n=4, 40%), BARC 3a (n=3, 30%), and BARC 3b (n=1, 10%). No BARC 3c, 4, or 5 events occurred. Clinically relevant bleeding (BARC ≥2) accounted for 80% of events (n=8). The timing of these bleeding events was periprocedural (≤30 days) in five patients (50%) and late (>30 days) in another five patients (50%). Periprocedural events included access-site hematoma (n=2), gastrointestinal bleeding (n=2), and hematuria (n=1); late events included gastrointestinal bleeding (n=3), epistaxis requiring intervention (n=1), and spontaneous ecchymosis with hemoglobin drop (n=1). Among the 13 patients with renal insufficiency (eGFR <60), the distribution of CKD stages was G3a (eGFR 45-59) in eight patients (three bleeding, 37.5%) and G3b (eGFR 30-44) in five patients (two bleeding, 40.0%). No patient had G4 or G5.

Risk factor analysis

Univariate logistic regression analysis (Table 2, with event counts) identified renal insufficiency (5/13 (38.5%) vs. 5/196 (2.6%); OR 23.87, 95% CI 5.73-99.49, p<0.001), age (per year, OR 1.17, 95% CI 1.05-1.30, p=0.003), and CHA₂DS₂-VASc score (OR 2.04, 95% CI 1.28-3.26, p=0.003) as factors associated with bleeding. Prior transient ischemic attack (TIA)/stroke (7/81 (8.6%) vs. 3/128 (2.3%); OR 3.94, 95% CI 0.99-15.71, p=0.052) and prior MI (1/3 (33.3%) vs. 9/206 (4.4%); OR 10.94, 95% CI 0.91-132.23, p=0.060) showed borderline associations. Sex, hypertension, diabetes, and other comorbidities showed no significant association.

**Table 2:** Univariate logistic regression analysis for bleeding events *p-values were calculated using univariate logistic regression; ^†^Absolute risk difference calculated as difference in observed proportions between categories (for binary variables) or per 10-unit increase in continuous variables; TIA: Transient ischemic attack; MI: Myocardial infarction

Variable	Events/n (%) in category	OR (95% CI)	p-value*	Absolute risk difference^†^
Age (per year)	_	1.17 (1.05-1.30)	0.003	+4.8% (per 10 years)
CHA₂DS₂-VASc (per point)	_	2.04 (1.28-3.26)	0.003	+3.2%
Renal insufficiency	5/13 (38.5%) vs. 5/196 (2.6%)	23.87 (5.73-99.49)	<0.001	+35.9%
TIA	7/81 (8.6%) vs. 3/128 (2.3%)	3.94 (0.99-15.71)	0.052	+6.3%
MI	1/3 (33.3%) vs. 9/206 (4.4%)	10.94 (0.91-132.23)	0.060	+28.9%
Hypertension	8/128 (6.3%) vs. 2/81 (2.5%)	2.63 (0.54-12.72)	0.228	+3.8%
Diabetes	3/34 (8.8%) vs. 7/175 (4.0%)	2.32 (0.57-9.47)	0.240	+4.8%
Sex (male vs. female)	5/114 (4.4%) vs. 5/95 (5.3%)	0.83 (0.23-2.94)	0.768	-0.9%

In standard multivariable logistic regression (including variables with p<0.1, TIA, MI, renal insufficiency, age, and CHA₂DS₂-VASc), renal insufficiency (adjusted OR (aOR) 16.67, 95% CI 2.51-110.93, p=0.004) and history of MI (aOR 107.49, 95% CI 3.86-2991.46, p=0.006) remained independently associated with bleeding, though confidence intervals were exceedingly wide (Table 3). These wide confidence intervals reflect the instability of traditional maximum-likelihood estimation in the setting of rare events.

**Table 3:** Standard multivariable logistic regression analysis (maximum likelihood) TIA: Transient ischemic attack; MI: Myocardial infarction

Variable	Category	Adjusted OR (95% CI)	p-value
TIA	Yes vs. no	5.51 (0.73-41.58)	0.098
MI	Yes vs. no	107.49 (3.86-2991.46)	0.006
Renal insufficiency	Yes vs. no	16.67 (2.51-110.93)	0.004
Age	Per year	1.13 (0.99-1.30)	0.076
CHA₂DS₂-VASc	Per point	1.15 (0.56-2.38)	0.704

**Figure 2:**
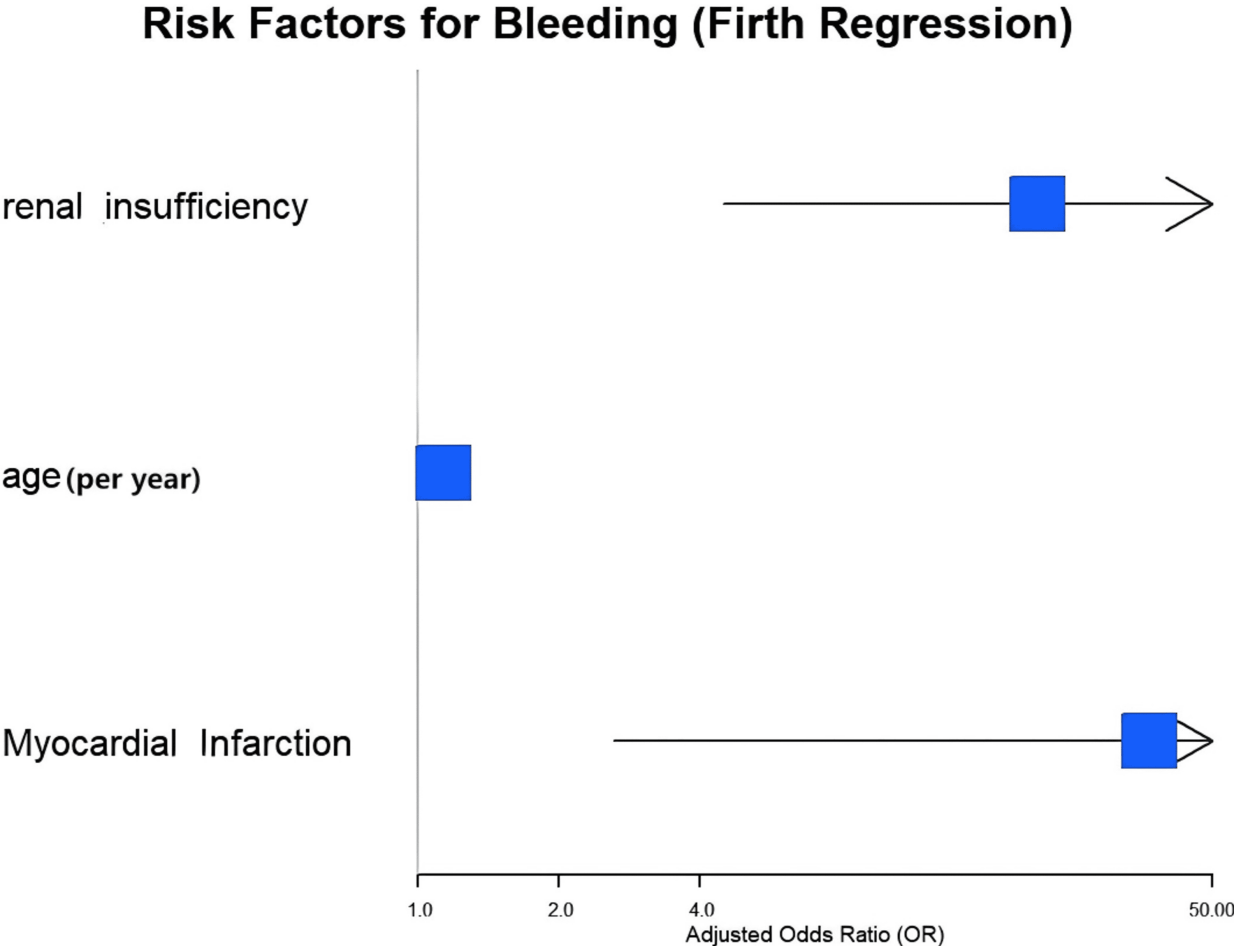
Forest plot showing aOR with 95% confidence intervals from Firth's penalized-likelihood regression analysis for independent predictors of bleeding events Age is presented per one-year increase. The vertical line represents the null effect (aOR=1.0). Points to the right of the line indicate increased bleeding risk. aOR: Adjusted odds ratio

To circumvent the instability associated with rare events, Firth's penalized-likelihood regression was undertaken (Table 4). This analysis, limited to three predictors (age, MI, renal insufficiency) based on EPV constraints, confirmed that age (aOR 1.13 per year, 95% CI 1.03-1.28, p=0.009; absolute risk difference per 10-year increase: +5.2%), history of MI (aOR 36.82, 95% CI 2.62-446.55, p=0.011; absolute risk difference: +41.3%), and renal insufficiency (aOR 21.16, 95% CI 4.50-106.48, p<0.001; absolute risk difference: +35.8%) were associated with bleeding events. A forest plot illustrating these multivariable regression results is presented in Figure 2.

**Table 4:** Multivariable Firth’s penalized-likelihood regression analysis *Absolute risk difference calculated as marginal effect from Firth model (difference in predicted probability); aOR: Adjusted odds ratio; MI: Myocardial infarction

Predictor	aOR	95% CI	p-value	Absolute risk difference*	Events/n (%) in category
Age (per year)	1.13	1.03-1.28	0.009	+5.2% (per 10 years)	-
MI	36.82	2.62-446.55	0.011	+41.3%	1/3 (33.3%)
Renal insufficiency	21.16	4.50-106.48	<0.001	+35.8%	5/13 (38.5%)

Model performance and validation

The apparent c-statistic for the three-predictor model was 0.86 (95% CI 0.77-0.94). After bootstrap optimism correction (200 replications), the optimism-corrected c-statistic was 0.82 (95% CI 0.71-0.91), suggesting modest overfitting but reasonable discrimination.

Collinearity assessment

Given conceptual overlap between age and CHA₂DS₂-VASc (which includes age as a component), we examined their correlation. Pearson's correlation coefficient was 0.72 (p < 0.001), and the variance inflation factor (VIF) was 5.2 when both were included in the same model, indicating substantial collinearity. For this reason, and to preserve EPV, CHA₂DS₂-VASc was not included in the primary multivariable model.

Sensitivity analyses

In the sensitivity analysis limited to clinically relevant bleeding (BARC ≥2, n=8), the direction and magnitude of associations were similar when it came to age (aOR 1.12 per year, 95% CI 1.02-1.26), renal insufficiency (aOR 19.8, 95% CI 3.9-101.2), and prior MI (aOR 32.4, 95% CI 2.1-498.3), though confidence intervals were wider due to even fewer events. Per influence diagnostics, the exclusion of the single patient with MI and bleeding (leave-one-out analysis) reduced the MI aOR to 18.4 (95% CI 1.2-289.6), indicating that this estimate is heavily influenced by a single observation and should be interpreted with particular caution. When CHA₂DS₂-VASc replaced age in the model (to assess collinearity impact), the aOR for CHA₂DS₂-VASc was 1.45 (95% CI 0.92-2.28, p=0.11), with minimal change in estimates for renal insufficiency and MI. The attenuated signal for CHA₂DS₂-VASc likely reflects its composite nature and overlap with other predictors.

EPV considerations

With 10 events, the inclusion of three predictors in the primary model yields an EPV of 3.3, below the conventional threshold of 10 recommended for reliable maximum-likelihood estimation. This EPV of 3.3, combined with the extreme sparsity of the MI predictor (only three patients total, one with bleeding), substantially increases the risk of overfitting and means that effect estimates, particularly the confidence intervals, should be interpreted as exploratory rather than definitive. The use of Firth regression mitigates, but does not eliminate, these concerns.

## Discussion

Principal findings

In this exploratory, single-center analysis of 209 NVAF patients with low baseline HAS-BLED scores (≤2) undergoing combined CA+LAAO, we identified three factors associated with post-procedural bleeding, namely renal insufficiency (eGFR <60 mL/min/1.73m²), history of MI, and advanced age. These associations persisted after adjustment using Firth regression to reduce small-sample bias and were directionally consistent in sensitivity analyses limited to clinically relevant bleeding (BARC ≥2). However, given the small number of bleeding events (n=10), the modest EPV ratio (3.3), and the extreme sparsity of the MI predictor, these findings should be considered hypothesis-generating rather than definitive. The wide confidence intervals, particularly for MI (aOR 36.82, 95% CI 2.62-446.55), underscore the imprecision of these estimates and the need for validation in larger cohorts.

Comparison with previous studies

Our findings align with and extend prior work on bleeding risk in AF patients undergoing interventional procedures. The association between renal impairment and bleeding risk is well-established in the context of anticoagulation [20,21] and percutaneous coronary interventions [22]. Chronic kidney disease affects multiple hemostatic pathways, including impaired platelet function, altered anticoagulant pharmacokinetics, and vascular calcification [23-25]. Our observation that even moderate renal impairment (CKD G3a, eGFR 45-59) was associated with bleeding risk, despite the exclusion of patients with severe impairment (eGFR <30), suggests that the threshold for heightened vigilance may be lower than traditionally appreciated. This finding is consistent with a recent meta-analysis of LAAO outcomes [26], which identified CKD as an independent bleeding predictor across the spectrum of renal function.

The strong association between prior MI and bleeding (aOR 36.82) is striking but must be interpreted cautiously, given that only three patients had a history of MI, of whom one experienced bleeding. The magnitude of this effect size is substantially larger than typically reported in anticoagulation studies [27] and likely reflects both the instability of estimates based on sparse data and potential unmeasured confounding. Biologically, prior MI may serve as a marker of systemic atherosclerosis and endothelial dysfunction [28], potentially rendering patients more vulnerable to bleeding under the dual stress of invasive procedures and antithrombotic therapy. However, with only one MI patient experiencing bleeding, this finding should be viewed as hypothesis-generating and requiring confirmation.

Advanced age as a bleeding risk factor is consistent with the study of anticoagulated populations [29]. Age-related changes include altered drug metabolism, increased vascular fragility, and higher comorbidity burden. Our finding that age remained associated with bleeding even within a cohort restricted to HAS-BLED ≤2 (where age contributes only 1 point if >65) suggests that the dichotomous age threshold in the HAS-BLED score may inadequately capture the continuous nature of age-related risk.

Reconsidering the HAS-BLED score in the procedural context

The HAS-BLED score was developed and validated to predict major bleeding during oral anticoagulation in ambulatory AF populations. Its application to the unique context of combined CA and LAAO, which involves vascular access, intraprocedural anticoagulation, myocardial injury from ablation, device implantation, and post-procedural antithrombotic therapy, represents a substantial extrapolation. Several factors may explain why patients with low HAS-BLED scores nevertheless experienced bleeding in our cohort.

First, the score's threshold-based approach may miss clinically important gradients. In HAS-BLED, age >65 contributes 1 point regardless of whether the patient is 66 or 86; similarly, 'renal disease' typically refers to dialysis, transplantation, or creatinine >200 μmol/L. Our data suggest that moderate renal impairment (eGFR 45-59) and incremental age beyond 65 convey bleeding risk not captured by the dichotomous scoring. Second, the score does not account for procedure-related risk. The combined CA and LAAO procedure creates a transient high-risk state through vascular access, heparinization, and myocardial tissue injury, which may interact with baseline patient characteristics to amplify bleeding risk. Third, the score assumes additive risk, whereas our findings raise the possibility of synergistic effects. The co-occurrence of renal impairment, prior MI, and advanced age may confer a multiplicative risk that simple additive scores cannot capture.

However, these observations should not be interpreted as definitive evidence of HAS-BLED inadequacy. Given the exploratory nature of our analysis and the small number of events, we cannot exclude the possibility that HAS-BLED performed as expected in this population. The observed bleeding rate of 4.8% over 30 months is within the range reported in contemporary LAAO registries [30], and whether the score would have performed better in a larger sample remains unknown.

Clinical implications

While our findings require validation before guiding clinical practice, they suggest several considerations for risk assessment in patients undergoing combined CA and LAAO. (1) Even moderate impairment (eGFR <60) may warrant enhanced vigilance of renal function, including consideration of more conservative antithrombotic regimens or closer post-procedural monitoring. (2) The incremental risk associated with age suggests that very elderly patients (e.g., >80 years) may merit individualized risk-benefit assessment beyond simple score-based classification. (3) Although based on extremely limited data, a history of MI may identify a subgroup with vascular vulnerability deserving of particular attention. We emphasize that these are hypothesis-generating observations, not practice recommendations. Any modification of periprocedural management based on these factors should occur only in the context of shared decision-making and acknowledgment of the uncertainty underlying these estimates.

Limitations

This study has several important limitations that temper the strength of its conclusions. First, the small number of events (n=10) is the most critical limitation. With only 10 bleeding events, statistical power is low, confidence intervals are wide, and the risk of type I and type II errors is high. The EPV ratio of 3.3 in our primary model is substantially below the recommended threshold of 10 for reliable logistic regression, increasing the risk of overfitting and model instability. While Firth regression reduces bias, it cannot create information where none exists.

Second, there is sparse data for MI. Only three patients had a history of MI, of whom one experienced bleeding. The odds ratio for MI is therefore heavily influenced by a single observation, as confirmed by influence diagnostics. This estimate should be regarded as particularly unstable and hypothesis-generating only.

Third, the single-center retrospective design introduces potential unmeasured confounding. We could not adjust for several factors that may influence bleeding risk, including post-procedural antithrombotic therapy (dosing, adherence, duration, and transitions); procedural details beyond energy source and device type; operator experience; and frailty or functional status. Post-procedural pharmacotherapy is a major potential confounder that was not adequately captured.

Fourth, selection bias may be present, as the requirement for complete follow-up data may have excluded patients with different outcomes. The single-center design limits generalizability to other populations and practice settings. Fifth is the range restriction. By design, the cohort was limited to patients with HAS-BLED ≤2. This restriction, while necessary to address the research question, limits the ability to assess the score's performance across its full range and may attenuate associations with predictors that contribute to the score. Sixth is multiple testing. The exploratory nature of the analysis involved multiple statistical comparisons without adjustment for multiplicity, increasing the risk of chance findings. And finally, no external validation was performed. Model performance was assessed through internal bootstrap validation, but external validation in an independent cohort is essential before any clinical application.

Future directions

These hypothesis-generating findings support several directions for future research. Larger multicenter prospective cohorts with adequate event numbers are required to enable reliable multivariable modeling and external validation. Procedure-specific risk models need to be developed to integrate baseline clinical factors with procedural variables and antithrombotic regimens. A detailed capture of antithrombotic therapy, including drug, dose, duration, adherence, and transitions, is necessary to enable adjustment for this critical confounder. Examination of renal function as a continuous predictor is essential to identify optimal risk thresholds. Novel biomarkers (e.g., markers of endothelial function and vascular calcification) need to be assessed to help improve risk prediction. A formal evaluation of the HAS-BLED score's performance in the procedural context needs to be made through direct comparison with procedure-specific models.

## Conclusions

In this exploratory, single-center analysis of NVAF patients with low baseline HAS-BLED scores undergoing combined CA and LAAO, renal insufficiency (eGFR <60), history of MI, and advanced age were associated with bleeding events. However, due to the small number of events (n=10), sparse data for key predictors, and absence of external validation, these findings should be considered hypothesis-generating rather than definitive. They highlight the need for larger, prospective multicenter studies to develop and validate procedure-specific risk models that may ultimately enable more individualized risk assessment in this population. Pending such validation, the HAS-BLED score remains a useful tool for initial risk stratification, but clinicians should be aware that patients classified as low risk may still experience bleeding, particularly in the presence of renal impairment or advanced age.
